# Mediterranean Diet and Cardiodiabesity: A Review

**DOI:** 10.3390/nu6093474

**Published:** 2014-09-04

**Authors:** Elena García-Fernández, Laura Rico-Cabanas, Nanna Rosgaard, Ramón Estruch, Anna Bach-Faig

**Affiliations:** 1 Mediterranean Diet Foundation, Barcelona 08021, Spain; E-Mails: elenagfdz@gmail.com (G.-F.E.); feinalaura@gmail.com (R.-C.L.); nanna2188@hotmail.com (R.N.); restruch@clinic.ub.es (E.R.); 2 Department of Health Sciences, Catalunya Open University (UOC), Barcelona 08018, Spain; 3 RETIC Healthy Nutrition in Primary Prevention of Chronic Diseases: Predimed Network (RD06/0045), Barcelona 08036, Spain; 4 Department of Internal Medicine, Hospital Clinic, August Pi Sunyer Biomedical Research Institute (IDIBAPS), Barcelona 08036, Spain; 5 Physiopathology of Obesity and Nutrition Center (CIBEROBN), Carlos III Health Institute, Madrid 28029, Spain; 6 Food and Nutrition Area, Barcelona Official College of Pharmacists, Barcelona 08009, Spain

**Keywords:** Mediterranean Diet, cardiodiabesity, systematic review, primary prevention, obesity, cardiovascular disease, diabetes mellitus, metabolic syndrome

## Abstract

Cardiodiabesity has been used to define and describe the well-known relationship between type 2 Diabetes Mellitus (T2DM), obesity, the metabolic syndrome (MetS) and cardiovascular disease (CVD). The objective of this study was to perform a scientific literature review with a systematic search to examine all the cardiovascular risk factors combined and their relationship with adherence to the Mediterranean Diet (MedDiet) pattern as primary prevention against cardiodiabesity in a holistic approach. Research was conducted using the PubMed database including clinical trials, cross-sectional and prospective cohort studies. Thirty-seven studies were reviewed: fourteen related to obesity, ten to CVD, nine to MetS, and four to T2DM. Indeed 33 provided strong evidence on the association between adherence to a MedDiet and a reduced incidence of collective cardiodiabesity risk in epidemiological studies. This scientific evidence makes the MedDiet pattern very useful for preventive strategies directed at the general population and also highlights the need to consider all these diet-related risk factors and health outcomes together in daily primary care.

## 1. Introduction

Cardiodiabesity is a hybrid term used to define and describe the well-known relationship between type-2 diabetes mellitus (T2DM), obesity, metabolic syndrome (MetS) and cardiovascular disease (CVD). Diabesity was first coined by former US Surgeon General C. Everett Koop [1] trying to explain that there were many risk factors to T2DM besides fatness. In 2008, in the British Journal of Cardiology [2], cardiologists started to mention the idea that diabetes should be viewed as a vascular disease from the outset. The term cardiodiabesity is not only useful to describe this framework but also to highlight the need to consider all the risk factors and health outcomes combined in clinical practice.

More than 347 million people worldwide have T2DM. It is foreseen that it will be the seventh leading cause of death by 2030 [3]. As with T2DM, obesity has reached epidemic proportions globally; from 1980 to 2008 the prevalence of obesity has doubled in the world, and at least 2.8 million people die each year as a result of being overweight or obese [4]. Furthermore, CVD was the cause of three out of ten deaths, accounting for nearly 17 million deaths in 2011 [5].

Based on this global framework, the European Society of Cardiology-European Association for the Study of Diabetes (ESC-EASD) established the close relationship between hyperglycemia and microvascular disease in their guidelines. The clustering of vascular risk seen in association with insulin resistance has led to the view that cardiovascular risk appears early, prior to the development of T2DM. This fact highlights that patients with T2DM should be managed as patients with established atherosclerotic disease [6].

Moreover, the guidelines of the National Institute for Health and Clinical Excellence (NICE) recommend treatment of dyslipidemia as a key modifiable risk factor in patients with T2DM and CVD [7,8]. Furthermore, it is well known that in central obesity, visceral adiposity tissue dysfunction leads to T2DM and CVD through increased peripheral insulin resistance due to high serum insulin concentrations. This clustering of vascular risk factors (insulin resistance, visceral or abdominal obesity and dyslipidemia) among other factors (hypertension) shapes the metabolic syndrome (MetS) [9,10]. These metabolic disturbances have an impact not only on glucose metabolism and the cardiovascular system, but also on gout and systemic inflammation, and predispose pancreatic tissue to pre-diabetes or diabetes itself [11]. Management of obesity and prevention of CVD and T2DM should be aimed at reducing the overall risk in a holistic health prevention and management approach.

There is substantial scientific evidence that diet plays an important role in the development of numerous chronic diseases. The Mediterranean Diet (MedDiet) has long been reported to be a prudent dietary pattern for non-communicable disease prevention [12,13,14,15,16], linked with nutritional adequacy [17] and has been promoted as a model of healthy eating based on its relation with preserving a good health status and quality of life [12,13], mainly through its favorable effects on cardiovascular risk factors and ultimately, by reducing cardiovascular morbidity and mortality [13].

This dietary pattern as described in the Mediterranean Diet pyramid [18] is generally characterized by a high consumption of plant foods (such as fruit, vegetables, legumes, nuts and seeds and cereals, preferably wholegrain); the seasonal choice of fresh and locally grown produce as far as possible; the presence of fruit as the main daily dessert and olive oil as the main source of dietary lipids; moderate consumption of dairy products (mainly cheese and yoghurt); low to moderate amounts of fish, poultry and eggs; consumption of red meat at a low frequency and in small amounts; and a moderate intake of wine during meals. Regarding the nutritional value of the MedDiet, this dietary pattern is low in saturated and trans fats, with an optimal nutritional quality due to the presence of healthy fats from olive oil, nuts and fish, as well as complex carbohydrates, micronutrients, antioxidants, non-nutritive factors and, furthermore, its abundant fiber and varied plant-based composition with sufficient protein intake of both plant and animal origin. The MedDiet pattern also gathers a proper ratio between the macronutrients, low energy density and low glycemic index meals [18,19].

The dietary pattern analysis approach has been imposed over the single nutrient or food approach [20,21]. Many MedDiet indexes [21] have been developed to study the relationship between the MedDiet pattern and different health parameters.

Not only the MedDiet has been stated as a health model, but also a cultural model after its recognition as an Intangible Cultural Heritage of Humanity by the United Nations Educational, Scientific and Cultural Organization (UNESCO) [22]. It is a culturally accepted and highly palatable dietary pattern that allows high compliance, food availability and affordability in the Mediterranean countries. Besides, the MedDiet pattern results in lower environmental footprints than the larger extended Western diet due to the greater emphasis on plant- over animal-derived products [23]. Among the scarce literature focused on evaluating the relationship between food costs and adherence to different food patterns [24], the MedDiet pattern is flexible and adjustable according to specific needs and preferences. Thus, the MedDiet is an extremely healthy, economically affordable and environmentally sustainable food model, especially in Mediterranean countries with a higher availability of MedDiet products. However, there has been a decrease in the adherence to the MedDiet in Mediterranean countries in the last decades [25,26,27], occurring in parallel to the “westernization” of the society (fast food, sedentary lifestyles, *etc.*). Although the relationship between these dietary and lifestyle changes and the increased prevalence of different illnesses has not been well established, there is a clear parallel trend between a higher prevalence of obesity, CVD or T2DM worldwide and the aforementioned changes in the dietary patterns of the population.

Although reviews of each specific disease such as obesity, T2DM or CVD in relation to the MedDiet have been published [13,15,28,29,30,31], up to now no review of the clinical trials, cross-sectional and prospective cohort studies has been performed to examine all these risk factors combined and their relationship with the MedDiet. Therefore, the aim of this review was to introduce the term cardiodiabesity and systematically summarize scientific evidence concerning the association between adherence to the MedDiet and the collective cardiodiabesity risk in all the studies.

## 2. Materials and Methods

The literature review was focused on prospective cohort, cross-sectional and clinical trial studies on the association between adherence to a MedDiet and cardiodiabesity, * i.e.*, MetS, T2DM, obesity and CVD as primary prevention outcomes. We excluded case-control design studies because of their related high potential selection bias.

A systematic search was conducted up to September 2013 through a computer-assisted published data search (PubMed; MEDLINE, National Library of Medicine, Bethesda, MD, USA). In PubMed, the MeSH terms used were “Mediterranean diet” along with other key words: “Diabetes Mellitus”, “Coronary Disease”, “Myocardial Ischemia”, “Heart Diseases”, “Metabolic Syndrome X”, and “Obesity”. The search was limited to human studies and restricted to articles in English. All studies with full text were considered. The initial search resulted in 740 articles. The search was then narrowed to include only articles examining the effect on the four outcomes (T2DM, obesity, CVD and MetS) as the main outcome and evaluating adherence to the MedDiet pattern as a whole. Studies focused on primary prevention of outcome events were selected. Additional publications were identified from references provided in original papers. The relevance of the studies was assessed with a hierarchical approach on the basis of title, abstract and the full manuscript.

**Figure 1:**
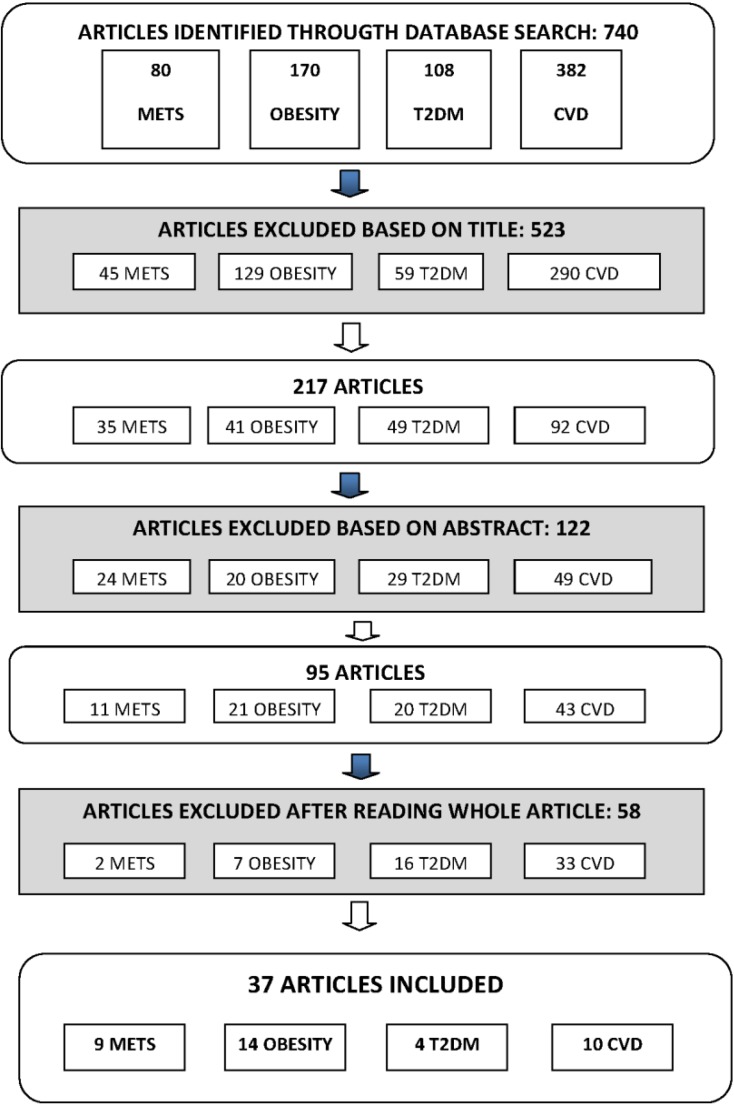
Flow-chart of the selection process. Abbreviations are as follows: METS, metabolic syndrome; T2DM, type 2 diabetes mellitus; CVD, cardiovascular disease.

A total of 740 articles were selected with these Mesh terms and then analyzed. The topic distribution was 80 for MetS, 170 for obesity, 108 for T2DM and 382 for CVD. Among the original research articles, 523 were excluded on the basis of the title and including reviews. From the total of 217 articles and on the basis of abstracts, 122 were excluded, resulting in 95 articles: 11 on MetS, 21 on obesity, 20 on T2DM and 43 on CVD. After reading the whole article, 37 were selected, resulting in the final inclusion of 14 studies on obesity, 9 on the MetS, 4 on T2DM and 10 on CVD (Figure 1).

## 3. Results

### 3.1. Characteristics of Study Sample

Most of the studies were from Mediterranean countries, specifically in Southern Europe, except for three conducted in the USA [32,33,34], two in Canada [35,36] and three in European countries outside the Mediterranean area [37,38,39]. Most had been conducted in both men and women, three studies only included females [35,38,40] and three involved children or adolescents [41,42,43]. All the studies were published between 2003 and 2013. The health status of the subjects varied among studies, but in the current review only includes studies on primary prevention.

The criteria of quality considered for the studies selected were the sample size, follow-up period and adjustment for potential confounders (demographic, anthropometric and traditional cardiovascular risk factors). Information regarding the methodology and weight-related results for these studies are summarized in Table 1. The sample sizes varied from 77 [35] and 497,308 [37] individuals. The potential confounders considered in the statistical analysis varied among studies, but the most common were age, sex, energy intake, smoking, physical activity and educational level, and also body mass index, marital status, family history of disease or pharmacological treatment.

To assess adherence to the MedDiet pattern, most of the studies included used the Mediterranean diet score established in 2003 by Trichopolou* et al.* [44,45], albeit with some modifications in many studies (detailed on Table 1). In addition, a considerable number of studies established their own score, for instance in the PREDIMED trial [46] the score for adherence to the MedDiet is based on a 14-item index.

**Table 1:** Characteristics of the studies included evaluating the relationship between the Mediterranean Diet and cardiodiabesity.

Author/Year of Publication	Country	Gender	Age (Years)	Follow-Up (Years)	*N*	Components in the MD Index	Main Outcome	Results	Confounders
Panagiotakos *et al.* 2005 [47]	Greece	♂1514 ♀1528	♂20–87; ♀20–89	1.25	3042	Score based on dietary pyramid [48]: (d) non refined cereals and products, fruits, olive oil, dairy products, wine; (w) fish, poultry, potatoes, olives, legumes, nuts, eggs, sweets; (m) red meat	T2DM	With 10 P increase score: OR 0.79 (95% CI 0.65–0.94), *p* < 0.05); ARR 1.3% (5.6%–4.3%); RRR 23.2% [(5.6%–4.3%)/5.6%]	Age, sex, BMI, WC, hc, education, financial status, smoking, family history of T2DM
Martínez-Glez *et al.* 2008 [49]	Spain	Men and women	20–90	4.4	13,380	MDS [45]: (+) fatty acid ratio, legumes, grains, fruits, vegetables, fish, wine; (−) dairy products or meat	T2DM	OR 0.41 (95% CI 0.19–0.87) (score 3–6); OR 0.17 (0.04–0.75) (score 7–9). With 2 P increase score RR 0.65, (0.44–0.95), *p* = 0.04	Sex, age, years of university education, TEI, BMI, PA, sedentary habits, smoking, family history of T2DM, and personal history of HT
Salas-Salvadó *et al.* 2011 [50]	Spain	Men and women	55–80	4	418	PREDIMED score 14 items [51]: olive oil, vegetables, fruit, fatty acid ratio, legumes, wine, fish, nuts; (−) sweets and carbonated beverages, red meat	T2DM	T2DM incidence MedDiet VOO 10.1% (95% CI 5.1–15.1), MedDiet nuts 11.0% (5.9–16.1), control group 17.9% (11.4–24.4). MedDiet VOO OR 0.49 (0.25–0.97) and nuts groups 0.48 (0.24–0.96). DM incidence was reduced 52% (27–86)	Age, sex, TEI, BMI, WC, PA, smoking status, fasting serum glucose, use of lipid lowering drugs, MedDiet score, and weight change during the study
Abiemo *et al.* 2013 [33]	USA	Men and women	45–84	6	5390	Score was created [45]: vegetables, whole grains, nuts, legumes, fruits, MUFA/SFA, red and processed meat, dairy products, fish, OH	T2DM	MeDiet score: lower baseline mean insulin levels, Q1: 5.8 (95% CI 5.6–6.0) μmol/L; Q5: 4.8 (95% CI 4.6–5.0) μmol/L; *p* < 0·0001. MedDiet not significantly related risk of T2DM *p* = 0.64	Demographic, physiological and behavioral characteristics
Panagiotakos *et al.* 2004 [52]	Greece	Men and women	>18	/	2282	Score created [53]: (d) cereals and products, vegetables, fruits, olive oil, dairy products; (w) fish, poultry, olives, legumes, nuts, potatoes, eggs, sweets; (m) red meat; (+m) wine and high MUFA/SFA ratio	MetS	OD 0.81 (95% CI, 0.68–0.976)	Age, sex, PA, education, smoking, BMI, diet in the past 12 months, TEI
Álvarez-León *et al.* 2006 [54]	Spain	Men and women	>18	/	578	10-Item score created [55]: cereal, fruits, vegetables, legumes, fish, nuts, alcohol intake from red wine, MUFA/SFA and dairy products	MetS	MD adherence not related to MetS prevalence; T2 OR 1.39 (95% CI, 0.75–2.59); T3 OR 1.37 (95% CI, 0.76–2.46)	Age, sex, PA, education, smoking, BMI, diet in the past 12 months, TEI
Tortosa *et al.* 2007 [56]	Spain	Men and women	20–90	6	2563	MDS [44]: (+) fatty acid ratio, legumes, grains, fruits, vegetables, fish, wine; (−) dairy products or meat.	MetS	MFP (95% CI) Age & sex adjusted score 3–5 OR 0.76 (0.40–1.45); score 6–9 OR 0.18 (0.06–0.56) *p* = 0.006; Multivariate-adjusted score 3–5 OR 0.80 (0.42–1.54); score 6–9 OR 0.20 (0.06–0.63)	Age, sex, PA, smoking, TEI
Salas-Salvadó *et al.* 2008 [57]	Spain	Men and women	♂55–80; ♀60–80	1	1224	PREDIMED score 14 items [51]: olive oil, vegetables, fruit, fatty acid ratio, legumes, wine, fish, nuts; (−) sweets and carbonated beverages, meat	MetS	MedDiet VOO group OR 1.3 (95% CI 0.8–2.1); MedDiet nuts group OR 1.7 (1.1–2.6)	Sex, age, baseline obesity status, and weight changes
Babio *et al.* 2009 [58]	Spain	Men and women	Mean 67	/	808	14-P questionnaire [59]: olive oil, vegetables, fruit, fatty acid ratio, legumes, wine, fish, nuts; (−) sweets and carbonated beverages, meat	MetS	↑MD score OR 0.44 (95% CI, 0.27–0.70)	Age, sex, PA, smoking, TEI
Bibiloni *et al.* 2011 [43]	Spain	Men and women	12–17	/	362	Score created [45]: high MUFA/SFA ratio; (+m) OH, high legume, cereals and roots, fruit, vegetables, and fish consumption, and low meat and milk consumption	MetS	Q2 MD 6.9%; Q4 5.9%; Glucose levels 0.89 (95% CI 0.40–1.90), triglyceride levels 0.72 (95% CI 0.31–1.77) and HDL-cholesterol levels 0.43 (95% CI 0.19–0.99)	Sex, age, parental educational level, parental socioeconomic status and PA
Rumawas *et al.* 2009 [34]	EEUU	Men and women	Mean 54	7	2730	Score created based on MSDPS [60]: whole-grain cereals, fruit, vegetables, dairy, wine, fish, poultry, olives/legumes/nuts, potatoes, eggs, sweets, meat, and olive oil	MetS	Q5 MSDPS ↓ incidence MetS (38.5% *vs.* 30.1%; *p* = 0.01) (95% CI) HOMA-IR Q5 3.16 (3.03–3.30) *p* = 0.02; fasting glucose 97.1 (96.3–98.0) *p* = 0.03; waist circumference 97.1 (96.7–97.6) *p* < 0.001; triglycerides 103 (99–107) *p* < 0.001; HDL-cholesterol 54.0 (53.1–55.0) *p* = 0.02	Height, weight, BMI, age, sex, smoking, multivitamin use, ERT, PA, TEI
Paletas *et al.* 2010 [61]	Greece	Men and women	44.4 ± 13.35	/	226	MDS [45]: (+) fatty acid ratio, legumes, grains, fruits, vegetables, fish, wine; (−) dairy products or meat	MetS	MetS components ↑ non-MD: MetS prevalence 27.3% MedDiet group, 60.2% HF group, and HC group 69.2% *p* = 0.006	Sex, BMI, TEI
Kesse-Guyot *et al.* 2013 [62]	France	Men and women	>18	6	3232	MDS [45], MSDPS [60] and MED [63]	MetS	↓ MetS: high MED OR 0.47 (95% CI 0.32–0.69) *p* = 0.001; high MDS OR 0.50 (95% CI 0.32–0.77) *p* = 0.03	Age, gender, education level, smoking status, PA, TEI, antidiabetic, antihypertensive and lipid lowering medications
Goulet *et al.* 2003 [35]	Canada	Women	30–65	0.25	77	MD: (+) cereals, fruit, vegetables, legumes, nuts and seeds, fish, olive oil, wine; (−) dairy products, chicken, eggs, sweets, red meat	OW and OB	↑ MD, ↓ BMI (mean week 0: 25.8 ± 3.9 kg/m^2^ to mean week 3 25.6 ± 3.8 kg/m^2^) *p* < 0.01; adh less weight (m0: 67.7 ± 11.9 kg to m3: 67.3 ± 11 kg) *p* < 0.01	
Schröder *et al.* 2004 [64]	Spain	♂1403 ♀1468	25–74	2	3162	MDS [44]: (+) cereals, vegetables, fruit, legumes, nuts, fish, red wine; (−) meat, high fat dairy products	OB	↑ MD, ↓ OB OR 0.61 *p =* 0.01; With 5-P MD, less BMI ♂ −0.43 kg/m^2^ *p =* 0.030; ♀ −0.68 kg/m^2^ *p =* 0.007	Age, TEI, educational level, smoking, LTPA, smoking and alcohol consumption
Trichopoulou *et al.* 2005 [65]	Greece	♂9612 ♀13,985	20–86	/	23,597	MDS [44]: (+) cereals, vegetables, fruit and nuts, legumes, fish, MUFA/SFA; (+m) OH; (−) meat and dairy products	OB	With 2 P increase score controlling TEI ♂ OR 0.08 (95% CI −0.03–0.20), ♀ OR −0.06 (95% CI −0.16–0.04); With 2 P increase score without controlling TEI ♂ OR 0.21 (95% CI 0.10–0.32), ♀ OR 0.05 (95% CI −0.04–0.15)	Age, years of schooling, smoking, PA, TEI
Shubair *et al.* 2005 [36]	Canada	♂265 ♀494	18–65	/	759	MDP score created: (+) fruit, vegetables, olive oil and garlic, fish and shellfish; (−) meats and poultry, high SFA, foods high in added sugar and low nutrients	OB	↑ MD, ↓ BMI (β-coefficient −0.186) *p = *0.027	Sex, education, income and marital status
Panagiotakos *et al.* 2006 [66]	Greece	♂1514 ♀1528	18–89	/	3042	MDS [48]: wholegrain cereals, vegetables, fruit, legumes, fish, olive oil, dairy products, chicken, nuts and seeds, olives, potatoes, eggs, sweets, meat, MUFA/SFA	OW and OB	With 5 P increased score OR 0.49 (95% CI 0.42–0.56) (OB & OW); ↑ MD, ↓ BMI (−4 kg) *p =* 0.001	Age, sex, PA, metabolism, educational level, smoking status
Mendez *et al.* 2006 [67]	Spain	♂10,589 ♀17,238	29–65	3.3	27,827	MDS [65]: (+) cereals, vegetables, fruit, legumes, fish, MUFA/SFA; (+m) OH; (−) meat	OB	S OW subjects ♀ OR 0.73 (95% CI 0.57–0.93), ♂ OR 0.71 (95% CI 0.55–0.93); NS OW subjects ♀ OR 0.99 (95% CI 0.78–1.25), ♂ OR 1.11 (95% CI 0.81–1.52)	Age, special diets related to obesity or related disorders, categorical activity index, education, center, height, parity, smoking status, winter season, follow-up time, health status and changes in lifestyle or health during follow-up
Andreoli *et al.* 2008 [40]	Italy	Women	25–70	0.33	47	MD moderately hypo-caloric: (+) vegetables, fruit, pasta, bread, legumes, fish, olive oil, red wine, meat, dairy products & PA program	Cardiovascular disease risk factors in obese women	↑ MD, ↓ weight (m0 80.4 ± 15.8 kg to m4 75.2 ± 14.7 kg) *p* < 0.001; ↑ MD, ↓ BMI (m0 30.7 ± 6.0 kg to m4 28.7 ± 5.6 kg) *p* < 0.001	
Panagiotakos *et al.* 2007 [68]	Cyprus	♂53 ♀97	65–100	/	150	MDS: wholegrain cereals, vegetables, fruit, legumes, fish, olive oil, dairy products, chicken, nuts, seeds, olives, potatoes, eggs, sweets, meat	OB	With 10 P increased score OR 0.83 (95% CI −0.25–0.12) *p* < 0.001 (having one additional risk factor); With 10 P increased score 12% lower OR OB *p* = 0.001	Age, sex, smoking habits, PA
Romaguera *et al.* 2009 [37]	10 European countries	Men and women	25–70	8	497,308	mMDS [44]: (+) vegetables, legumes, fruits, nuts, cereals, fish and seafood, MUFA/SFA; (+m) OH; (−) meat, dairy products	OB	↑ MD, ↓ WC, for a given BMI ♂ 20.09 (95% CI 20.14–20.04), ♀ 20.06 (95% CI 20.10–20.01); Northern European countries ♂ 20.20 (95% CI 20.23–20.17), ♀ 20.17 (95% CI 20.21–20.13)	Age, educational level (categorical), PA, smoking status, height, menopausal status
Beunza *et al.* 2010 [69]	Spain	Men and women	Mean 38	5.7 ± 2.2	10,376	MDS [45]	OW and OB	↑ MD, ↓ weight 20.059 kg/year 95% CI 20.111–20.008 kg/year, *p* = 0.02; lowest risk weight gain 4-year 0.76 (95% CI 0.64–0.90)	Age, sex, baseline BMI, PA, sedentary behaviors, smoking, between-meals snacking, TEI
Romaguera *et al.* 2010 [70]	10 European countries	Men and women	25–70	5	373,803	rMED [71]: (+) vegetables, legumes, fruit, nuts, cereals, fish and seafood, olive oil; (+m) OH; (−) meat, dairy products	OW & OB	↑ rMED, ↓ weight gain 5-year 20.16 kg (95% CI 20.24, 20.07 kg); less likely to OW or OB 10% (95% CI 4%–18%)	Sex, age, baseline BMI, follow-up, educational level, PA, smoking status, menopausal status, TEI, misreporting of TEI
Lazarou *et al.* 2010 [41]	Cyprus	Men and women	Mean 10.7 ± 0.98	/	1140	KIDMED [72]	OB	↑ KIDMED, ↓ likely to OW/OB 80% (95% CI 0.041–0.976)	Age, gender, parental obesity status, parental educational level, dietary beliefs and behaviors
Farajian *et al.* 2011 [42]	Greece	Children	10–12	/	4786	KIDMED [72]	OW & OB	NS NW (score mean 3.70 ± 2.26) & OW/OB (score mean 3.62 ± 2.26), *t*-test 1.08, *p* = 0.28	Age, BMI, waist, body fat mass
Martínez-Glez *et al.* 2012 [73]	Spain	Men and women; 57%♀	55–80	/	7447	PREDIMED [51]	OB	With 2 P increase score ♀ WHtR OR −0.65 (95% CI −0.87–0.48), BMI OR −0.37 (95% CI −0.50–0.24), WC OR −0.92 (−1.26–0.58); ♂ WHtR OR −0.58 (95% CI −0.79–0.38), BMI OR −0.25 (95% CI −0.37–0.13), WC OR −0.93 (95% CI −1.29–0.58)	Age, smoking, diabetes status, HT status, PA, educational level, marital status, center, TEI
Trichopoulou *et al.* 2003 [45]	Greece	Men and women	20–86	Median 3.7	22,043	9 Unit MDS [44]: (+) vegetables, legumes, fruits, nuts, cereals, fish; (+m) OH; (−) meat, poultry, dairy products	Fatal CHD events	↑ MD, ↓ CHD mortality HR 0.67 (95% CI 0.47–0.94)	Sex, age, years of education, smoking status, WtHR, BMI, TEI, energy-expenditure score
Knoops *et al.* 2004 [39]	11 European Countries	Men and women	70–90	12	2339	mMD [45]: MUFA/SFA, legumes, nuts, seeds, grains, fruit, vegetables and potatoes, meat, dairy products, fish	All-cause and cause specific mortality	↑ MD score, ↓ mortality: all causes 0.77 (0.68–0.88), CHD 0.61 (0.43–0.88), CVD 0.71 (0.58–0.88) (95% CI)	Other diet and lifestyle factors, sex, age at baseline, BMI, and study population (SENECA *vs.* FINE).
Panagiotakos *et al.* 2007 [74]	Greece	Men and women	18–89		3042	Own score [48]: (+) non-refined cereals, fruit, vegetables, legumes, potatoes, fish, olive oil; (−) meat, poultry, full fat dairy products; (+m) OH	CVD risk factors	With 10 P increase score, 10-year follow up 4% lower CHD risk (±0.1%, *p* < 0.001); ↑ MD, ↓ 10-year follow up CHD risk (rho −0.7, *p* < 0.001)	No assessment forpotential confounding since all analyses were unadjusted
Buckland *et al.* 2009 [71]	Spain	Men and women	29–69	Mean 10.4	41,078	r-MED [45]: (+) fruits, nuts, seeds, vegetables (no potatoes), legumes, cereals, fresh fish, olive oil; (−) meat, dairy products	CHD events	↑ MD score, ↓ CHD risk, *Z* ¼ 0.60 (95% CI 0.47–0.77); with 1 P increase score, 6% reduced risk CHD (95% CI 0.91–0.97)	Age, BMI, educational level, smoking status, PA, TEI, T2DM, hyperlipidemia, HT
Fung *et al.* 2009 [38]	U.K.	Women	38–63	20	74,886	aMED [45]: (+) vegetables (excluding potatoes), fruits, nuts, whole grain, legumes, fish, MUFA/SFA; (+m) OH; (−) meat	Risk of stroke	Q5 ↓, risk CHD RR 0.71 (95% CI 0.62–0.82), *p* = 0.0001 for CHD; ↓ risk stroke RR 0.87 (95% CI 0.73–1.02), *p* = 0.03; Q5, ↓ CVD mortality ♀ RR 0.61 (95% CI 0.49–0.76), *p* = 0.0001	Age, smoking, BMI, menopausal status, postmenopausal hormone use, TEI, multivitamin intake, OH intake, family history, PA, aspirin
Gardener *et al.* 2011 [32]	EEUU (NY)	Men and women	69 ± 10	9	2568	Own MD Score [45]: dairy, meat, fruit, vegetables (excluding potatoes), legumes, cereals (refined and whole), fish	CHD events	Q5 score (1) HR 0.75 (95% CI 0.56–0.99) *p* = 0.04; (2) HR 0.80 (0.60–1.06) *p* = 0.10. With 1P increase score (1) HR 0.94 (0.89–1.00), (2) HR 0.95 (0.90–1.01)	(1) Age, sex, ethnicity, education, PA, TEI, smoking; (2) model 1 and T2DM, HT, hypercholesterolemia, history self-reported cardiac disease
Martínez-Glez *et al.* 2011 [75]	Spain	Men and women	Mean 38	4.9	13,609	9-Point score [45]: (+) MUFA/SFA, legumes, cereals, fruits, nuts, vegetables, fish; (+m) OH; (−) total dairy products, meat	CHD events	↑ MD, ↓ CVD risk HR 0.41 (95% CI 0.18–0.95); with 2 P increase score, total CVD HR 0.80 (95% CI 0.62–1.02), CHD HR 0.74 (95% CI 0.55–0.99)	Age, sex, TEI, family history CHD, smoking, PA, baseline BMI, HT or use of medication, aspirin, baseline T2DM, baseline dyslipidaemia
Menotti *et al.* 2012 [76]	Italy	Men and women	45–64	5	1139	MAI [77]: (+) cereals, legumes, potatoes, vegetables, fresh dry fruit, fish, wine, virgin olive oil; (−) milk, cheese, meat, eggs, animal fats and margarines, sweet beverages, sugar	CHD events	20-Year follow-up lnMAI HR 0.74 (95% CI 0.56–0.98) *p =0.0338*; 40-year follow-up lnMAI HR 0.79 (95% CI 0.65–0.96) *p = 0.0168*	Age, cigarette smoking, systolic blood pressure, serum cholesterol, PA, BMI
Guallar-Castrillón *et al.* 2012 [78]	Spain	Men and women	29–69	8–12; median 11	40,757	Own score	CHD events	MD score HR Q2 0.77 (95% CI 0.61–0.98), Q3 0.64 (95% CI 0.50–0.83), Q4 0.56 (95% CI 0.43–0.73), Q5 0.73 (95% CI 0.57–0.94)	Age, sex, BMI, WC, educational level, smoking, PA at work, home and leisure time, T2DM, HT, hypercholesterolemia, cancer, oral contraceptives, menopausal status, hormone replacement therapy, TEI
Estruch *et al.* 2013 [46]	Spain	Men and women	55–80	4.8	7447	PREDIMED [51]	CHD events	MD VOO HR 0.70 (95% CI 0.54–0.92); MD nuts HR 0.72 (95% CI 0.54–0.96)	Sex, age, BMI, cardiovascular-risk-factor status, baseline adherence MedDiet

MD, Mediterranean Diet; ♂ men; ♀ women; MUFA/SFA, ratio of monounsaturated to saturated fat; Meat, meat and meat products; Dairy, milk and dairy products; (+), positive components; (−), negative components; (+m), components positive in moderation; (d) daily; (w) weekly; (m) monthly; adh, adherence; HR, hazard ratio; OH, alcohol consumption; PA, physical activity; TEI, total energy intake; WC, waist circumference; hc, hip circumference; HT, hypertension; CI, confidence interval; OR: odds ratio; BMI, body mass index; T2DM, type 2 diabetes mellitus; ARR, absolute risk reduction; RRR, relative risk reduction; Q, quartile; P, points; *p* for trend; MetS, metabolic syndrome; MDS, Mediterranean diet score; MED, Mediterranean score; MSDPS, Mediterranean style-dietary pattern score; HC, high-carbohydrate diet group; HF, high-fat diet group; T, tertile; mMDS, modified-Mediterranean Diet Score; rMED, Mediterranean Diet Score; LTPA, leisure-time physical activity; OW, overweight; NW, normal weight; S, significant; NS, not significant; WHtR, waist-to-height ratio; CHD, coronary heart disease; ACS, acute coronary syndrome; MAI, Mediterranean Adequacy Index; DM, diabetes mellitus; VOO, virgin olive oil; CVD, cardiovascular disease; 10 European countries (Denmark, France, Germany, Greece, Italy, the Netherlands, Norway, Spain, Sweden, the United Kingdom).

### 3.2. Association between the MedDiet and Cardiodiabesity

**Figure 2:**
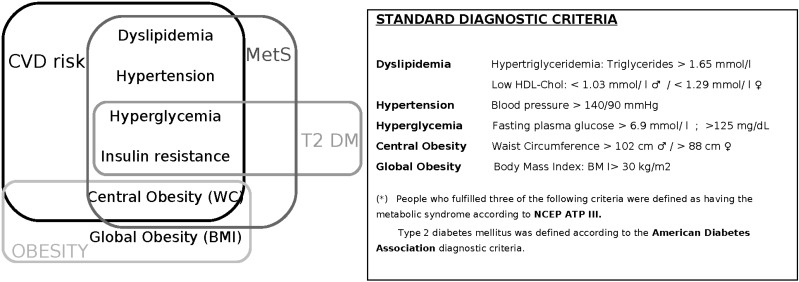
Diet-related Cardiodiabesity diagnostic parameters. Abbreviations are as follows: METS, metabolic syndrome; T2DM, type 2 diabetes mellitus; CVD, cardiovascular disease; WC, waist circumference; BMI, body mass index.

Out of the 37 studies, 33 reported a significant association between adherence to MedDiet and reduced incidence and prevalence of cardiodiabesity, but in four there was no association between MedDiet, T2DM, obesity, MetS and CVD [33,37,54,65]. Figure 2 describes the overall diet-related cardiodiabesity parameters and cardiovascular risk factors used in the different studies reviewed.

Regarding the association between adherence to the MedDiet pattern and T2DM, Abiemo* et al.* [33] reported that a greater consistency with a MedDiet style was cross-sectionally associated with lower serum insulin concentrations among non-diabetics and with lower blood glucose before adjustment for obesity but not with a lower incidence of DM. However, three out of the four studies selected found that a higher MedDiet score was predictive of a lower incidence of T2DM [47,49,50]. The greatest relative reduction in T2DM risk (52%) was observed in the PREDIMED nutritional intervention trial in subjects at high CVD risk who showed high adherence to the traditional MedDiet [50]. The incidence of T2DM was reduced by 35% in the cohort study of Martínez-González* et al.* [49] in association with a two-point increase in the Trichopoulou score, adjusting for confounders. Panagiotakos* et al.* [47] described that a 10-unit increase in the diet score was associated with 21% lower odds of T2DM, while the combination of greater adherence to the MedDiet and light physical activity reduced the risk of diabetes by 35% after adjustment for various factors.

The impact of the MedDiet pattern on the prevalence of the MetS was evaluated in five cross-sectional studies selected in this review [43,52,54,58,61]. In all these studies, the MetS criteria were defined according to the National Cholesterol Education Program (NCEP) Adult Treatment Panel (ATP) III [79], which includes hypertriglyceridemia, low HDL-cholesterol, hypertension, hyperglycemia and central obesity. People who fulfilled three or more of these conditions were defined as having MetS. Alvarez-León* et al.* [54] concluded that MedDiet adherence was not related to MetS prevalence even though some components of the MedDiet showed a protective effect on the MetS and its components. The ATTICA study [52] determined that the odds ratio of having MetS decreased when the participants consumed a MedDiet and reported little-to-moderate physical activity. Other cross-sectional studies reported significant inverse associations between high adherence to the MetDiet and the prevalence of MetS criteria in elderly men with high CVD risk [58], in adolescents [43] and in overweight/obese subjects [61], respectively. Moreover, the three cohort studies selected, with a follow-up of 6 to 7 years, found the lowest incidence of MetS in subjects with the highest adherence to the MedDiet [34,56,62]. In addition, the overall prevalence of MetS at the first year assessment of the PREDIMED cohort showed a higher reduction in participants who consumed a MedDiet supplemented with mixed nuts compared with patients given advice to follow a low-fat diet [57]. It should be noted that in some studies individual components of the MetS, especially hyperglycemia, were not always significantly affected [56,57,62].

Most of the studies selected showed that the prevalence of overweight, obesity and central obesity was inversely associated with the MedDiet score. Classified according to study type, three cohort studies [67,69,70] reported that adherence to a MedDiet pattern was significantly associated with a reduced weight gain, and that the risk to develop overweight and obesity was also less likely. Specifically, Mendez* et al.* [67] reported a decrease in obesity with MedDiet adherence, albeit only being significant among the overweight population. All the interventional dietary studies [35,40,73] found that adherence to a MedDiet significantly decreased weight/BMI and, specifically, abdominal obesity. Regarding the cross-sectional studies, the EPIC-PANACEA study by Romaguera* et al.* [37] found that higher adherence to the MedDiet was significantly associated with lower abdominal adiposity for a given BMI, measured by waist circumference, while the MedDiet was not significantly associated with general obesity (BMI). Tripocholou* et al.* [65] did not find any association between MedDiet adherence and weight either. In contrast, five of the seven cross-sectional studies [36,41,64,66,68] found that greater adherence to a MedDiet had a significantly negative association with overweight/obesity. The strongest association was reported in the ATTICA study by Panagiatakos *et al.* [66] who found that individuals with high MedDiet adherence were 51% less likely to have both central adiposity and obesity, after adjustment for potential confounders. Note that in the CYKIDS study by Lazarou* et al.* [41] although adherence to the MedDiet was inversely associated with childhood obesity, when physical activity was taken into account, this relationship became less significant. Furthermore, children with higher adherence to a MedDiet reported to practice higher physical activity levels [42].

All the studies on the primary prevention of CVD showed a statistically significant association between the MedDiet pattern and the incidence of CVD [32,38,39,45,46,71,74,75,76,78]. In the PREDIMED trial, the largest randomized trial aimed to assess the effect of a dietary intervention using MedDiet on CVD outcomes; Estruch* et al.* [46] found that among persons at high cardiovascular risk, a MedDiet supplemented with extra-virgin olive oil or mixed nuts reduced the incidence of major cardiovascular events by 30%. Regarding cohort studies, adherence to the MedDiet was associated with a lower risk of coronary heart disease (CHD) in the large Spanish cohort of the EPIC study [71,75,78]. This MedDiet association has been assessed by either by using *a priori* [71,75] or *a posteriori* scales [78] during a median follow-up period of 10 years. The longest follow-up period was conducted in the Italian cohort study by Menotti* et al.* [76] in which individuals with a higher adherence to the MedDiet showed a protective effect against the occurrence of fatal CHD events at 20 and 40 years (CHD mortality reduction of 26% and 22%, respectively). Additionally, Tripochoulou* et al.* [45] (from the Greek cohort of the EPIC study) reported that the MedDiet was associated with a significant reduction in mortality due to CHD. Moreover, an inverse association with greater adherence to a MedDiet was observed for all-cause mortality and cause-specific death such as CHD and CVD [39], lower risk of stroke [38] and other vascular events, such as myocardial infarction and vascular death [32].

## 4. Discussion

This review is focused on the potential beneficial role of a MedDiet pattern for overall primary cardiodiabesity prevention taking into account the most solid, epidemiological and updated scientific evidence available. Several reviews have previously reported the beneficial effect of the MedDiet on obesity [30], T2DM, MetS [80] and CVD [81] risk factors. In this review, cardiodiabesity is considered as a term that embodies the overall diet-related diseases, which are the first causes of death worldwide, as well as others that affect and worsen health conditions.

Of the 37 studies reviewed in this report, 33 provided strong evidence on the association between adherence to a MedDiet and CDV, T2DM, MetS and obesity. Moreover, results from the large scale randomized intervention study, the PREDIMED trial, have pointed out that after 5 years of intervention among high-risk persons who were initially free of CVD, a MedDiet (with no caloric restriction) supplemented with one daily serving of mixed nuts or up to 50 mL of extra-virgin olive oil reduced the incidence of three major cardiovascular events (cardiovascular death, myocardial infarction and stroke) [46] by 30%, suggesting a higher protective effect against cardiovascular risk factors with a MedDiet compared to a low-fat diet, with the added strength of higher compliance among the followers of the MedDiet [46,51]. This protective effect of the MedDiet observed appears early after starting the dietary change and is similar to the effects of statin and antihypertensive drug treatments but without side effects. In addition, the Portfolio diet described by Jenkins and colleagues, which comprised four key components as foods rich in soluble fiber, soy protein, plant sterols and almonds, reported a 29% reduction in LDL-cholesterol comparable to that observed with a small dose of a statin [82].

To increase the quality and level of scientific evidence, in this review we included prospective observational cohorts and clinical trials. Case-control studies were excluded because of the related bias as mentioned. Nevertheless, the results among the studies reviewed were not completely consistent. Romaguera* et al.* [37] and Trichopoulou* et al.* [65] found no significant relationship between adherence to a MedDiet and BMI, but they did find a relationship with a lower waist circumference for a given BMI in both genders. Abiemo* et al.* [33] reported that higher adherence to MedDiet was associated with lower insulin concentrations even though it did not decrease the incidence of T2DM, and the MeDiet also showed protective effects on some MetS components although the Alvarez* et al.* [54] study did not fulfill the conditions that—according to NCEP-ATPIII criteria—more than three components have to be present to diagnose MetS. Thus some studies reported no clear association between adherence to a traditional MedDiet and main outcomes although they did find positive associations with intermediate risks factors [33,37,54,65]. The variability of the associations observed and the strength between adherence to a MedDiet and cardiodiabesity could also be linked to several aspects and limitations inherent to differences between studies and samples. Similarly, other potential limitations were adjustments for different confounders and indexes used as described in the table. Moreover, specifically designed studies should be carried out to assess these outcomes simultaneously in order to determine the synergy of interventions in several health parameters involved in cardiodiabesity.

According to the American Public Health Association (APHA), in the USA obesity has about 152 billion dollars/year and 73 billion dollars/year in direct and indirect (lost productivity, absenteeism,* etc.*) costs, respectively [83]. In the current economic context, variables related to health management and the cost-benefit ratio are particularly relevant to health policies and clinical protocols, and, thus, to action guidelines. Current recommendations highlight the importance of dietary counseling in clinical practice. Even though the MedDiet is a diet with a high fat intake, and fat sources are derived primarily from monounsaturated oils, in their last guideline on T2DM, pre-diabetes, and CVD [6], the European Society of Cardiology together with the European Association for the Study of Diabetes group (ESC/EASD) stated, that the MedDiet is an acceptable alternative to the traditionally proposed DASH diets, which are mostly prescribed in clinical practice today. Besides, ESC/EASD support the consideration that T2DM treatment and prevention should be firmly based on complex and non-pharmacological therapies [84]. The ADA 2013 nutrition therapy recommendations provide a summary of strong scientific evidence supporting the effectiveness of nutrition therapy across the continuum of diabetes management [84], which has reported that non-drug treatment is as effective as hypoglycemic drug-treatment, involving a mean reduction of 1.0%–1.5% in HbA1c [84]. The role of nutrition in the prevention of CVD has been extensively reviewed [85,86,87] and strong evidence of the influence of dietary factors in atherogenesis both directly and through effects on traditional risk factors, such as lipid profile, blood pressure or plasma glucose concentration has been reported [88]. Thus, management of obesity and prevention of CVD and T2DM should be aimed at reducing the overall risk of cardiodiabesity in a holistic manner.

Despite the beneficial effects of the MedDiet, there are discrepancies among nutrition experts because of the high-fat content of this diet (up to >40% of total energy intake), which is in conflict with the usual recommendation to follow a low-fat diet in order to avoid overweight/obesity and to prevent CHD [89,90,91]. A recent meta-analysis [92] argued that current evidence does not clearly support cardiovascular guidelines that encourage high consumption of polyunsaturated fatty acids and low consumption of total saturated fats. However, it should be remarked that the PREDIMED trial demonstrated with the highest level of scientific evidence that the MedDiet is a useful tool to prevent CVD end-points in high-risk subjects [46]. On the other hand, the higher palatability, acceptance and compliance with MedDiets in comparison with low-fat diets should also be taken into account [93].

This review shows that most of the diet-related risk factors of cardiodiabesity may be preventable through healthy lifestyles and specifically with a healthy dietary pattern. Note that all the studies included took into account the physical activity of the participants but no study undertook active intervention controlling of this variable. Thus, all evidence on the reported beneficial effect was mainly due to the overall composition of the dietary pattern, without other lifestyle changes. Preventive medicine should be part of current and future public health strategies in the global context of high rates of chronic diseases around the world. Nowadays, important cultural and economic changes are affecting the population lifestyle, and consequently its health. In recent decades, there has been a strong social transformation that has led to poorer food habits, a decline in traditional customs and a change in the MedDiet pattern [48,91,94]. Promotion of a healthy lifestyle can be used for the prevention or treatment of several factors contributing to cardiodiabesity and lead to the avoidance of progression and the need for pharmacological interventions. The Spanish Society of Family and Community Medicine (SEMFYC) agrees that there is sufficient evidence on the benefits of the dietary advice carried out by trained personnel on the prevention of several chronic diseases, but, on the other hand, there is little evidence on the effectiveness of dietary advice in primary care, probably due to inadequate dietary assessment and other factors. Thus, the dietary advice targeted at apparently healthy or low-risk asymptomatic individuals should be simple and easy to comply with [95]. The new MedDiet pyramid [18] is the result of an international consensus based on the latest scientific evidence on nutrition and health, and provides key elements for the quantitative and qualitative selection of foods. This new MedDiet pyramid is meant for use and promotion without any restrictions, and the nutritional recommendations based on a MedDiet pattern may lead to the prevention of chronic diseases, especially if dietary recommendations take into account individual preferences, thereby ensuring long-time adherence. In this sense, the MedDiet could be translated into daily clinical primary care practice in the setting of public health promotion programs. In order to facilitate this step and achieve greater compliance with dietary advice, the development of the following points should be taken into account:

(i) To develop simple, objective and useful tools to determine the degree of patient adherence to the MedDiet pattern for its application in the clinical setting and general practice.
(ii) To establish consensual recommendations about the MedDiet pattern and psychological approaches to eating habits and lifestyle improvements. Moreover, it is important to foster knowledge of and accessibility to this dietary pattern to health professionals, particularly in primary care where preventive medicine is of greatest, albeit not exclusive, significance. Multidisciplinary approaches and strategies are needed to increase compliance to treatments and even dietary and lifestyle interventions [96].

## 5. Conclusions

Most of the reviewed studies provided strong evidence on the association between adherence to a MedDiet and CDV, T2DM, MetS and obesity, remarking the relationship between all these interconnected illnesses and supporting the term cardiodiabesity. The results of the current review of epidemiological and clinical trial studies support the role of the MedDiet in the prevention of cardiodiabesity. The prevention of cardiodiabesity by good adherence to the MedDiet is supported by the latest most solid scientific evidence, and further by its low environmental footprints and economic accessibility in Mediterranean countries. Furthermore, the high palatability of the MedDiet makes this dietary pattern very useful for preventive strategies applied to the general population in primary care medicine for optimal collaborative management of these patients.
